# Changes in nutritional composition and bioactivity of fermented cereals under *Pleurotus ostreatus* solid-state fermentation

**DOI:** 10.3389/fnut.2026.1919003

**Published:** 2026-09-08

**Authors:** Tetiana Zhuk, Dmytro Bakhlukov, Valeriia Nikitenkova, Manuela Dorin, Tetiana Zaichenko, Victor Barshteyn, Mustafa Sevindik, Tetiana Krupodorova

**Affiliations:** 1 Faculty of Chemical Technology, National Technical University of Ukraine “Igor Sikorsky Kyiv Polytechnic Institute”, Kyiv, Ukraine; 2 Institute of Food Chemistry and Food Biotechnology, Justus Liebig University Giessen, Giessen, Germany; 3 Department of Plant Food Products and Biofortification, Institute of Food Biotechnology and Genomics of the National Academy of Sciences of Ukraine, Kyiv, Ukraine; 4 Department of Biology, Faculty of Engineering and Natural Sciences, Osmaniye Korkut Ata University, Osmaniye, Türkiye

**Keywords:** bioactive compounds, enzymatic and antioxidant activity, fermentation, oyster mushroom, rice, wheat

## Abstract

**Introduction:**

The edible and medicinal mushroom *Pleurotus ostreatus* is increasingly recognized as a functional ingredient, not only as a source of fungal biomass but also as a biological fermenter capable of transforming cereal substrates. This study evaluated time-dependent changes in nutritional composition and bioactivity during *P. ostreatus* solid-state fermentation of cereals.

**Methods:**

Wheat and rice were fermented with *P. ostreatus* for 42 days at 25 °C. Antioxidant activity, enzyme activities, total phenolic content (TPC), proximate composition, and amino acid composition were evaluated using standard analytical methods. Antioxidant activity, enzyme activities, and TPC were determined for both substrates. Based on the stepwise evaluation of cereal substrates, proximate composition and amino acid analyses were subsequently performed for wheat.

**Results:**

Visual assessment showed that both substrates were fully colonized by day 21. Bioactive compounds and enzyme activities were monitored from days 21 to 42. Enzyme activities showed distinct substrate- and time-dependent patterns: in wheat, esterase and laccase activities peaked on day 35 and peroxidase activity was numerically highest on day 42 but did not differ significantly from day 35, whereas in rice, esterase activity was highest on day 42, and laccase activity on day 28, and peroxidase activity on day 21. Aryl-alcohol oxidase activity remained low in both substrates. TPC and DPPH radical scavenging activity increased during fermentation, with both reaching their highest values on day 28 in wheat. In rice, TPC was highest during the late fermentation stage (days 35–42), whereas DPPH radical scavenging activity peaked on day 35. In wheat, true protein content increased from 6.43 to 18.65 g/100 g DM during the fermentation period, accompanied by an increase in ash content and a gradual decrease in carbohydrate content, while fat content remained relatively stable. The amino acid profile also changed during fermentation, with significant increases in histidine, glutamic acid, and lysine. True protein and total amino acid content continued to increase until day 42, day 28 provided the most balanced essential amino acid profile.

**Conclusion:**

The optimal fermentation period depends on the desired characteristics of the final product and highlight the importance of monitoring fermentation dynamics to optimize cereal solid-state fermentation.

## Introduction

Cereals are major dietary staple worldwide, providing essential carbohydrates, proteins, and essential micronutrients for a large portion of the global population. However, the nutritional quality and bioavailability of raw grains are often limited by anti-nutritional factors, such as phytic acid, tannins, and enzyme inhibitors, which restrict mineral absorption and protein digestibility (1). In recent years, microbial fermentation has emerged as a key bioprocessing technique to overcome these limitations. Solid-state fermentation (SSF), in particular, is carried out on solid substrates with low moisture content, closely resembling the natural growth conditions of many microorganisms. Compared with submerged fermentation, SSF requires less water while enabling efficient structural modification of plant matrices and high product yields. During SSF, endogenous and microbial enzymes break down complex macromolecules, transforming low-value or underutilized grains into more digestible, value-added products (1, 2).

Microbial fermentation using bacteria, yeasts, and filamentous fungi has been widely applied to improve the nutritional and technological properties of cereal grains. Fermentation alters the contents and composition of carbohydrates, proteins, major lipid fractions, and dietary fiber while also improving technological characteristics such as gelatinization, swelling, and rheological properties (3). These changes are strongly influenced by fermentation parameters, including substrate composition, inoculum selection, and process conditions.

Legumes (soybeans, lentils, beans, peas), cereals (rice, wheat, buckwheat, millet, barley, oats, sorghum, and corn), and pseudocereals (quinoa) have been used as substrates for SSF (4–6). Among these substrates, wheat (7–9), various rice cultivars, including Riceberry (10–12), Tatar buckwheat (13), and soybean (9, 14) have been studied most extensively.

While traditional food fermentations have relied on filamentous fungi, such as *Aspergillus*, *Rhizopus*, or *Mucor* species, modern research is increasingly shifting focus toward Basidiomycete fungi (5). Higher Basidiomycetes have highly efficient lignocellulolytic enzyme systems that are uniquely capable of degrading complex agricultural fibers and lignin components (15). Edible and medicinal macromycetes are of particular interest for cereal fermentation because they efficient degrade plant cell wall components while simultaneously synthesizing biologically active metabolites, including immunomodulatory polysaccharides (16, 17). Substrate transformation during SSF is driven by the coordinated action of extracellular oxidative and hydrolytic enzymes produced by *Pleurotus ostreatus*. Accordingly, esterase, laccase, peroxidase, and aryl-alcohol oxidase were selected as representative enzymes to monitor the dynamics of substrate bioconversion during fermentation. Their combined activities provide complementary information on the progression of substrate bioconversion and the associated oxidative and hydrolytic processes during fermentation. However, the vast majority of studies on macromycete-driven SSF have focused either on lignocellulosic substrates or on a limited selection of agro-industrial residues, leaving the metabolic changes in edible whole grains largely underexplored.

SSF with edible and medicinal macromycetes has attracted increasing attention for the bioprocessing of cereals and pseudocereals. Recent studies using species of the genus *Grifola* (7), *Agaricus bisporus*, *Helvella lacunosа*, *Fomitiporia yanbeiensi* (4), *Auricularia auricula*, *Hericium erinaceus* (13), *Ganoderma lucidum* (8, 13), *Pleurotus eryngii* (10), *P. ostreatus* (9, 14), and *P. sapidus* (11), have shown that fungal fermentation can improve the nutritional and functional characteristics of grain substrates. Reported changes include increased antioxidant activity (4, 7, 8, 10, 14), greater accumulation of bioactive compounds and improved nutritional composition (8–12, 14) as well as modifications to the technological and sensory properties of cereal-based products (6). These studies suggest that fungal SSF is a promising strategy for improving the nutritional value of cereal raw materials and expanding their potential food applications. Among the fungi used in cereal fermentation, *P. ostreatus* has emerged as a particularly suitable species due to its robust growth, efficient lignocellulolytic activity, and broad biotechnological applicability. In this context, investigating the changes in nutritional composition and bioactivity of cereals during *P. ostreatus* SSF can support the development of cereal-based fermented foods with improved nutritional and functional properties.

Previous studies have shown that solid-state fermentation with *P. ostreatus* improves the nutritional quality of cereal and legume substrates by modifying their composition, reducing antinutritional factors, and increasing the phenolic content and antioxidant activity (9, 12). Fermentation has also been reported to enhance the prebiotic properties of pigmented rice under specific experimental conditions (14). However, comparative information on the time-dependent biochemical changes associated with *P. ostreatus* fermentation of different cereal substrates remains limited. This study aimed to compare the biochemical and functional changes in wheat and rice during *P. ostreatus* fermentation and to characterize the nutritional composition of fermented wheat at the selected fermentation time.

## Materials and methods

### Fungal culture and maintenance

*P. ostreatus* (Jacq.) P. Kumm. strain 80, corresponding to the commercial strain P80 (Italspawn, Italy), was kindly supplied by the commercial producer of exotic and medicinal mushrooms “Kingdom of Mushrooms” (Kyiv, Ukraine). The strain is deposited in the IBK Mushroom Culture Collection under accession number IBK 2462 (18). The fungal culture was stored at 4 °C on malt extract agar slants. To maintain viability (every 3 months) and for preparation as seed culture, this strain was transferred in Petri dishes with glucose peptone yeast agar medium (GPYA, pH 6.0) with the following composition per liter: 25.0 g glucose, 2.0 g yeast extract, 3.0 g peptone, 1.0 g K_2_HPO_4_, 1.0 g KH_2_PO_4_, 0.25 g MgSO_4_·7H_2_O, and 20.0 g agar. Petri dishes containing GPYA medium were inoculated with a single mycelial disc (8 mm diameter) under sterile conditions and incubated at 25 °C for 7 days.

### Solid state fermentation

Rice and wheat were purchased from the local food market in Kyiv, Ukraine. The cereal substrates were pre-cooked in an industrial steam-jacketed kettle (KPE-100, Ukraine) at 100 ± 2 °C for 10–15 min (rice) or 30 min (wheat). Portions of the substrates (150 g) were transferred into 500-mL glass jars, covered with aluminum foil, and sterilized in an autoclave at 121 °C for 30 min. The substrate loading provided an appropriate solid-to-headspace ratio for fungal cultivation.

Under sterile conditions, the substrates were inoculated with three agar plugs (10 mm in diameter) taken from a 7-day-old culture of *P. ostreatus* previously grown on commercial potato dextrose agar (Difco, USA) at 25 ± 2 °C. For each cereal substrate, three independent fermentation jars were prepared from a single commercial batch for each cereal substrate. Wheat and rice were purchased from different manufacturers, and each cereal type was represented by a single commercial batch. Cultivation was carried out in a thermostatically controlled room at 25 ± 2 °C in the dark. Samples were collected after 21, 28, 35, and 42 days of fermentation. Based on our previous observations using the same *P. ostreatus* strain and comparable cereal substrates, the substrate appeared fully colonized after approximately 21 days under these cultivation conditions, as assessed by visual inspection of mycelial growth (6). The first sampling point was day 21. A non-inoculated substrate was prepared and analyzed before fermentation (day 0) as the initial control for comparison with the fermented samples.

### Preparation of enzyme extracts

Whole fermented culture samples (500 mg dry weight) were rehydrated in 5 mL of 0.1 M potassium phosphate buffer (pH 7.0) and incubated for 2 h at 24 °C with shaking (600 rpm) in an HLC Biotech Heating ThermoMixer (DITABIS AG, Pforzheim, Germany). After incubation, the suspensions were centrifuged at 4,000 × g for 5 min, and the resulting supernatants were collected and used for enzyme activity assays.

### Aryl-alcohol oxidase (AAO) activity assay

Aryl-alcohol oxidase activity was determined spectrophotometrically using *p*-anisyl alcohol as substrate (19). The supernatant prepared as described above was used for the assay. Measurements were performed in triplicates using 96-well microplates. A reaction mixture of 165 μL was prepared, of which 150 μL was transferred into each well for measurement. Depending on the sample absorbance caused by the coloration of the enzyme extracts, the reaction mixture contained 75 or 95 μL of 50 mM phosphate buffer (pH 7.0) and 40 or 20 μL of enzyme sample, respectively. Lower supernatant volumes were used for highly colored samples to minimize background absorbance and measurement interference. The mixture also contained 25 μL of catalase (1 mg/1 mL aqueous solution, from bovine liver (2,000–5,000 units/mg protein), Sigma, St. Louis, USA) and 25 μL of 50 mM substrate [dissolved in 50 mM potassium phosphate buffer (pH 7.0)]. The deactivated control was prepared by mixing the enzyme extract with the 50 mM phosphate buffer and heating the mixture at 80 °C for 10 min. After cooling, the assay was performed under the same conditions as for the active samples. Formation of *p*-anisyl aldehyde (*ε*290 = 12,372.67 M^−1^ cm^−1^) was followed at 290 nm using a Synergy 2 reader (BioTek, Friedrichshall, Germany). Enzyme activity was monitored for 30 min at 30 °C. One unit of activity is defined as the amount of enzyme that converts 1 μmol substrate per minute under the stated conditions. The enzyme activity was expressed as U L^−1^ of enzyme extract. The activity was calculated according to the following equation:

$$\text{Activity}\;(U\;L^{-1})=\frac{\mathrm{\Delta E}\cdot V_{\text{total}}\cdot F}{V_{\text{sample}}\cdot \varepsilon_{\mathrm{nm},\mathrm{pH}}\cdot d},$$

where Δ*E* is the difference between the mean extinction of the active and deactivated sample; *V*_total_ is the reaction volume in the microplate well; *F* is the dilution factor (1); *V*_sample_ is the volume of enzyme extract used in the assay; *ε* is the molar extinction coefficient at the respective wavelength and pH; and *d* is the optical path length (0.48 cm).

### Esterase activity assay

Esterase activity was determined spectrophotometrically using *p*-nitrophenyl acetate as substrate (20). The enzyme extract prepared as described above was used for the assay. Measurements were performed with a total reaction volume of 200 μL containing 130 or 140 μL of 80 mM potassium phosphate buffer (pH 7.0), 20 or 10 μL sample, and 50 μL of 3.5 mM substrate prepared by mixing one part of a 17.5 mM substrate [solution in 96% EtOH with four parts of 80 mM potassium phosphate buffer (pH 7.0)]. Formation of *p*-nitrophenol (*ε*405 = 9,850 M^−1^ cm^−1^) was followed at 405 nm using a Synergy 2 reader (BioTek, Friedrichshall, Germany) (21). Enzyme activity was monitored for 10 min at 30 °C. Enzyme activity was calculated as described above and expressed as U L^−1^ of enzyme extract.

### Laccase activity assay

Laccase activity was determined spectrophotometrically using ABTS as substrate (22). The enzyme extract prepared as described above was used for the assay. Measurements were performed in triplicate using 96-well microplates with a total reaction volume of 200 μL containing 80 or 90 μL of 150 mM sodium acetate buffer (pH 3.5), 20 or 10 μL sample, 50 μL of water solution of catalase (6 U mL^−1^, from bovine liver (2,000–5,000 units/mg protein), Sigma, St. Louis, USA) and 50 μL of 2.0 mM substrate [dissolved in 150 mM sodium acetate buffer (pH 3.5)]. Formation of the resulting ABTS radical cation (*ε*420 = 43,200 M^−1^ cm^−1^) was followed at 420 nm using a Synergy 2 reader (BioTek, Friedrichshall, Germany) (22). Enzyme activity was monitored for 30 min at 30 °C. Enzyme activity was calculated as described above and expressed as U L^−1^ of enzyme extract.

### Peroxidase activity assay

Peroxidase activity was determined spectrophotometrically using ABTS as substrate (22). The enzyme extract prepared as described above was used for the assay. Measurements were performed in triplicate using 96-well microplates with a total reaction volume of 200 μL containing 80 or 90 μL of 150 mM sodium acetate buffer (pH 3.5), 20 or 10 μL sample, 50 μL of 1.4 mM H_2_O_2_ in distilled water, and 50 μL of 2.0 mM substrate [dissolved in 150 mM sodium acetate buffer (pH 3.5)]. Formation of the resulting ABTS radical cation (*ε*420 = 43,200 M^−1^ cm^−1^) was followed at 420 nm using a Synergy 2 reader (BioTek, Friedrichshall, Germany). Enzyme activity was monitored for 10 min at 30 °C. Enzyme activity was calculated as described above and expressed as U L^−1^ of enzyme extract.

### Preparation of extracts

Mycelium-covered cereal samples were collected on days 21, 28, 35, and 42, then dried at 60 °C in a Snol-58/350 drying cabinet (UMEGA, Lithuania) until their moisture content was below 10%. Dried samples were separately ground into fine powder using an electric grinder (VHC-150, Kyiv, Ukraine). For extraction, 1 g of each fermented cereals was mixed with 10 mL of ethyl acetate (EtOAc) and incubated at room temperature on an orbital shaker (Santarius, Göttingen, Germany) at 100 rpm for 72 h. Ethyl acetate was selected because it preferentially extracts low- and medium-polarity phenolic compounds while minimizing the extraction of highly polar matrix components, including water-soluble polysaccharides, which may interfere with spectrophotometric assays. After extraction, the sample were centrifuged at 9,000 rpm at 4 °C for 10 min (Eppendorf MiniSpin, Hamburg, Germany), and the resulting supernatants were collected for subsequent analyses (total phenolic content and antioxidant activity).

### Assay of total phenolic content (TPC)

TPC of the extracts was quantified using the Folin–Ciocalteu method (23), employing gallic acid as the standard. The procedure involved mixing 1 mL of the extract sample with 0.5 mL of Folin–Ciocalteu reagent (diluted 1:10 with deionized water), followed by the addition of 4 mL of sodium carbonate solution (75 g·L^−1^). The resulting mixture was vortexed for 15 s (Fisher Vortex Genie 2, New York, USA) and incubated at 40 °C for 30 min. The absorbance was measured at 765 nm using a UV–Vis spectrophotometer (UV-1800 PC, Shanghai, China). A reagent blank containing all assay components except the extract was prepared. Results were expressed as milligrams of gallic acid equivalents per gram of dry weight (mg GAE/g d.w.).

### Assay of antioxidant activity

The antioxidant activity of the extracts was determined using the 1,1-diphenyl-2-picrylhydrazyl (DPPH) assay (24). The assay procedure involved mixing 100 μL of each extract with 2,900 μL of DPPH solution (120 μM in MeOH). The resulting mixtures were incubated in the dark at 37 °C for 30 min. Absorbance was measured at 517 nm, with methanol serving as the blank. A control containing DPPH solution without the extract was included. DPPH radical scavenging activity (%) was calculated according to the following equation:

$$\text{Inhibition}\;(\%)=\frac{\left(A_{\text{control}}-A_{\text{samples}}\right)}{A_{\text{control}}}\times 100,$$

where *A*_control_ is the absorbance of the control (mixture of MeOH and DPPH reagent without fungal extract). *A*_sample_ is the absorbance of the sample (mixture of fungal extract and DPPH reagent).

### Proximate chemical analysis

Moisture content was quantified using a Moisture Analyzer MA35 (Sartorius, Göttingen, Germany). True protein values were calculated from the amino acid composition as the sum of amino acid residues. Crude fat was quantified after acid hydrolysis and petroleum ether extraction using a Soxtherm SOX416 rapid extraction system (C. Gerhardt GmbH & Co. KG, Königswinter, Germany). Ash content was determined gravimetrically from the residue remaining after incineration at 550 °C.

All proximate composition data were expressed on a dry matter (DM) basis, and before carbohydrate content was calculated by difference using the following equation:

Carbohydrates(%)=100−(true protein+crudefat+ash)

### Amino acids content

Amino acid composition was determined as described by Ahlborn et al. (25). Briefly, dried and finely ground samples (25–30 mg) were hydrolyzed in 6 M HCl containing phenol (1 g L^−1^) at 110 °C for 24 h. After hydrolysis, the samples were neutralized, adjusted to pH 2.2, diluted with citrate buffer, and filtered through a 0.45 μm membrane filter. For the determination of cysteine and methionine, samples were subjected to performic acid oxidation before acid hydrolysis. Tryptophan was determined separately following alkaline hydrolysis with 5 M NaOH containing phenol (1 g L^−1^) at 110 °C for 24 h. Amino acids were quantified using an S433 amino acid analyzer (Sykam, Eresing, Germany) equipped with an LCA K13/Na column, an LCA K04/Na pre-column, and controlled with ChromStar software (version 7).

To determine the true protein content, amino acid residues (AAres), representing amino acids incorporated into proteins after the loss of water during peptide bond formation, were calculated from the amino acid composition, which was measured by the amino acid analyser. A sample-specific nitrogen-to-protein conversion factor (NPCF) was then calculated as the ratio of AAres to the total nitrogen content. True protein content was calculated by multiplying the total nitrogen content by the corresponding NPCF. The calculated NPCF values are provided in the Supplementary material. This approach accounts for the actual amino acid composition of each sample rather than applying the conventional conversion factor of 6.25 for crude protein.

$$\text{NPCF}=\frac{\text{AAres}}{\text{Total nitrogen}}$$

True protein=Total nitrogen×NPCF

Protein quality was evaluated by calculating the amino acid score (AAS), chemical score (CS), essential amino acid index (EAAI), and biological value (BV) using the corresponding equations:

$$\mathrm{AAS}=\frac{{\%}^{"}\text{amino}\;\mathrm{aci}d^{"}\;\text{in test protein}}{\%\text{corresponding amino acid in the reference protein}},$$

where FAO/WHO is the reference protein. The FAO/WHO (1973) reference protein was used to maintain consistency with previous studies on fermented proteins and protein quality evaluation. Since many earlier studies investigating amino acid chemical scores of fermented foods employed this reference pattern, its use facilitates direct comparison of the present results with the existing literature.

CS=AAS×100

$$\begin{array}{l} \mathrm{EAA}\;\text{Index}\\ =\sqrt[{8}]{CS_{\mathrm{Ile}}\times CS_{\mathrm{Leu}}\times CS_{\mathrm{Lys}}\times CS_{\mathrm{Met}+\mathrm{Cys}}\times CS_{\mathrm{Phe}+\mathrm{Tyr}}\times CS_{\mathrm{Thr}}\times CS_{\mathrm{Trp}}\times CS_{\mathrm{Val}}} \end{array}$$

BV=1.09×EAAI−11.7

### Statistical analysis

All experiments were conducted using three independent biological replicates, except for the amino acid analysis, which was performed using two independent biological replicates. Technical replicates were averaged before statistical analysis. Data are presented as mean ± standard deviation (SD).

Enzyme activities, TPC, and DPPH radical scavenging activity were analyzed using two-way analysis of variance (ANOVA), with substrate type, cultivation time, and their interaction as fixed factors. Enzyme activities were measured after 21, 28, 35, and 42 days of cultivation, whereas total phenolic content and DPPH radical scavenging activity were evaluated at days 0, 21, 28, 35, and 42.

Before ANOVA, the assumptions of normality and homogeneity of variances were evaluated using the Shapiro–Wilk and Levene’s tests, respectively. When significant effects were detected, Tukey’s HSD test was used for pairwise comparisons. Statistical significance was accepted at *p* < 0.05.

Principal component analysis (PCA) and hierarchical clustering were carried out using the mean values of three independent biological replicates in the ClustVis online platform.^1^ No data transformation was applied before analysis. The data were mean-centered and scaled to unit variance. PCA was performed using singular value decomposition (SVD) with imputation, whereas hierarchical clustering was based on Euclidean distance and average linkage.

## Results

### Colonization of cereal substrates and enzyme activities

Сolonization of the cereal substrate is an important indicator of mycelial growth. It reflects the development of the fungus and is closely related to its metabolic activity, particularly the production of extracellular enzymes. Based on visual assessment of mycelial growth, the substrate appeared fully colonized after 21 days of cultivation. This cultivation period allowed the detection of potential issues such as uneven mycelial growth, contamination, or signs of mycelial autolysis. The period from day 21 to day 42 was selected to evaluate the enzymatic activity after most of the readily available nutrients in the substrate had been consumed. At this stage, the fungus shifts toward the production of secondary metabolites and enzymes involved in the further degradation of the remaining substrate.

**Figure 1:**
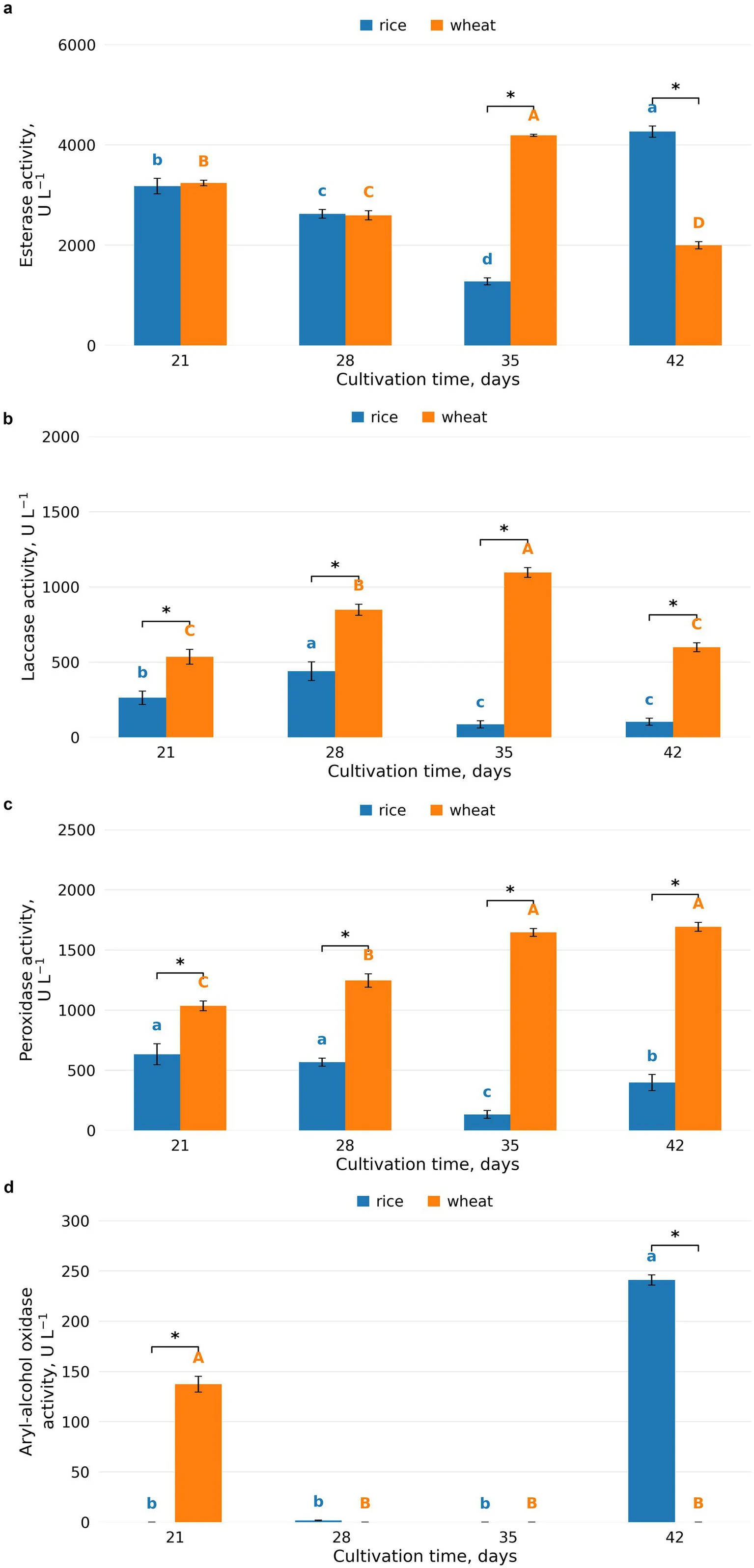
Effect of cultivation time on the enzymatic activity (U L^−1^ of enzyme extract) of cereals fermented with *Pleurotus ostreatus*: **(a)** Esterase activity, **(b)** Laccase activity, **(c)** Peroxidase activity, **(d)** Aryl-alcohol oxidase activity (AAO). Samples were analyzed after 21, 28, 35, and 42 days of cultivation. Error bars represent mean ± SD of three independent biological replicates (*n* = 3). Different lowercase letters (blue) indicate significant differences among rice cultivation times, whereas different uppercase letters (orange) indicate significant differences among wheat cultivation times, (*) indicates a significant difference between rice and wheat at the same cultivation time (Tukey’s HSD, *p* < 0.05).

Standard 96-well plate assays were employed to quantify the activities of esterase, laccase, peroxidase, and aryl-alcohol oxidase in crude extracts prepared from fermented cereal samples collected between days 21 and 42 of cultivation. Enzyme activities varied with cereal type and cultivation time (Figure 1).

The activity of esterase changed non-linearly during cultivation (Figure 1a). Esterase activity remained similar on days 21 and 28 in both cereals, after which it increased significantly, peaking on day 35 in wheat and day 42 in rice. Laccase activity increased from day 21 to day 28 of cultivation in rice, whereas in wheat the increase continued significantly until day 35 (Figure 1b). Wheat showed a peak in laccase activity on day 35, whereas rice reached its highest activity on day 28. Peroxidase activity in fermented cereals was slightly higher than laccase activity (Figure 1c). Fermented wheat exhibited a clear linear pattern of peroxidase activity, suggesting a steady enzymatic response that may be related to substrate availability or changes in fungal metabolism. In contrast, fermented rice showed a wave-like pattern of peroxidase activity. This suggests that enzyme production changed over time, possibly reflecting shifts in fungal physiology or substrate utilization. Peroxidase activity in wheat was numerically highest on day 42 but did not differ significantly from day 35, whereas rice reached its maximum activity at day 21. Aryl-alcohol oxidase activity was the lowest among the enzymes analyzed. In wheat, AAO activity was detected only on day 21. In rice, AAO activity reached its highest level on day 42, whereas no significant differences were observed among days 21, 28, and 35 (Figure 1d). The observed differences indicate that enzyme activities were influenced by the cereal substrate. These results highlight the importance of selecting an appropriate substrate when developing biotechnological applications based on ligninolytic enzymes.

### Changes in total phenolic content and antioxidant activity

**Figure 2:**
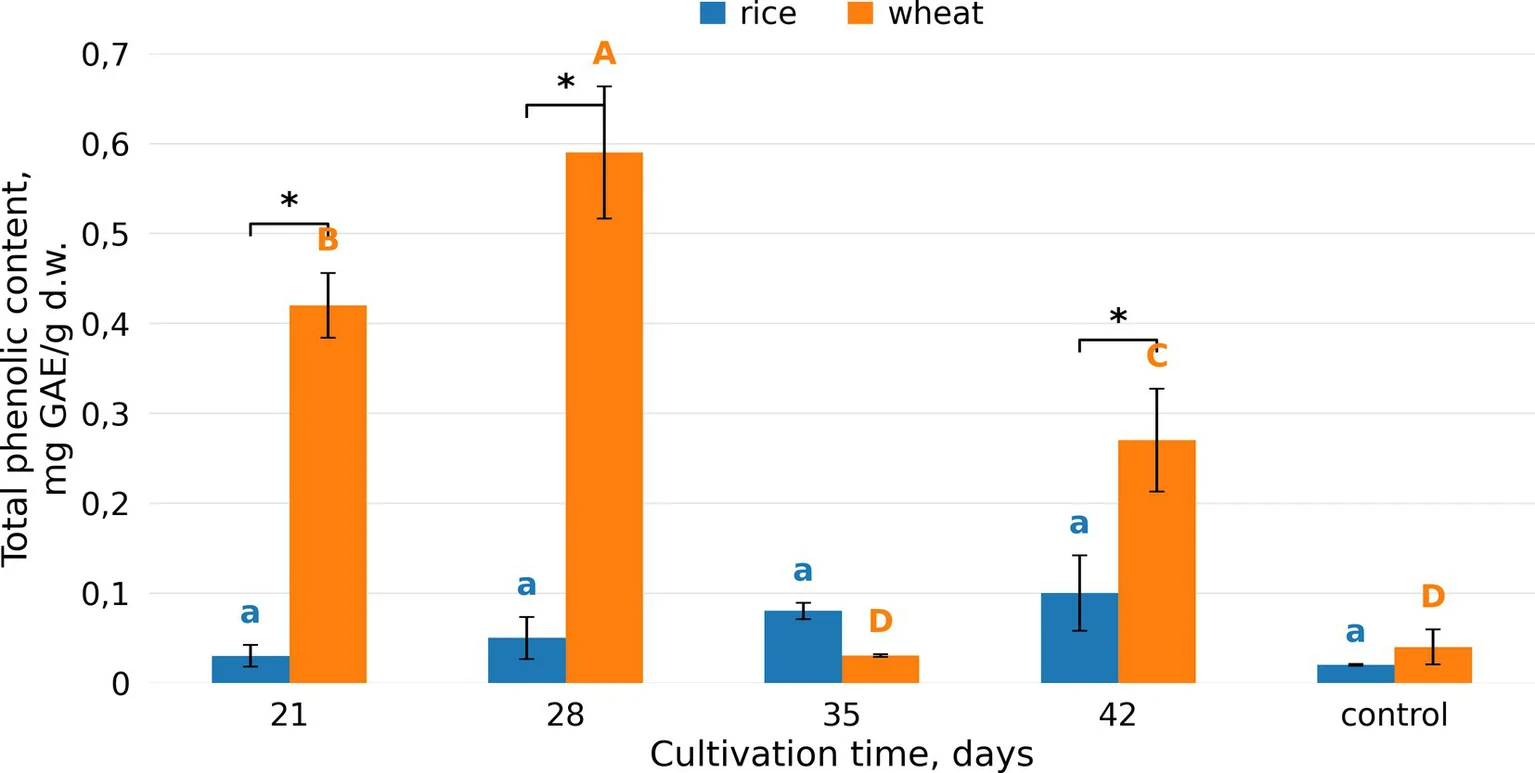
Effect of cultivation time on total phenolic content (mg GAE g^−1^ d.w.) of cereals fermented with *Pleurotus ostreatus* and control (unfermented cereals). Error bars represent mean ± SD of three independent biological replicates (*n* = 3). Different lowercase letters (blue) indicate significant differences among rice cultivation times, whereas different uppercase letters (orange) indicate significant differences among wheat cultivation times, (*) indicates a significant difference between rice and wheat at the same cultivation time (Tukey’s HSD, *p* < 0.05).

A comparative analysis of cereals fermented with *P. ostreatus* revealed distinct phenolic accumulation patterns (Figure 2). Rice showed a more gradual and stable increase in TPC but reached substantially lower levels than wheat. In contrast, fermented wheat exhibited higher TPC than rice on days 21, 28 and 42, whereas rice showed higher values on day 35. The highest TPC was recorded in wheat on day 28. In rice, TPC reached its highest values during the late stage of fermentation, with no significant difference between days 35 and 42. Compared with the unfermented control, TPC in wheat was significantly higher on days 21, 28, and 42, whereas no significant increase was observed on day 35. In rice, TPC showed a gradual increase during fermentation and reached its highest values at the late stage (days 35–42). However, no significant differences were observed either among the cultivation times or relative to the unfermented control.

**Figure 3:**
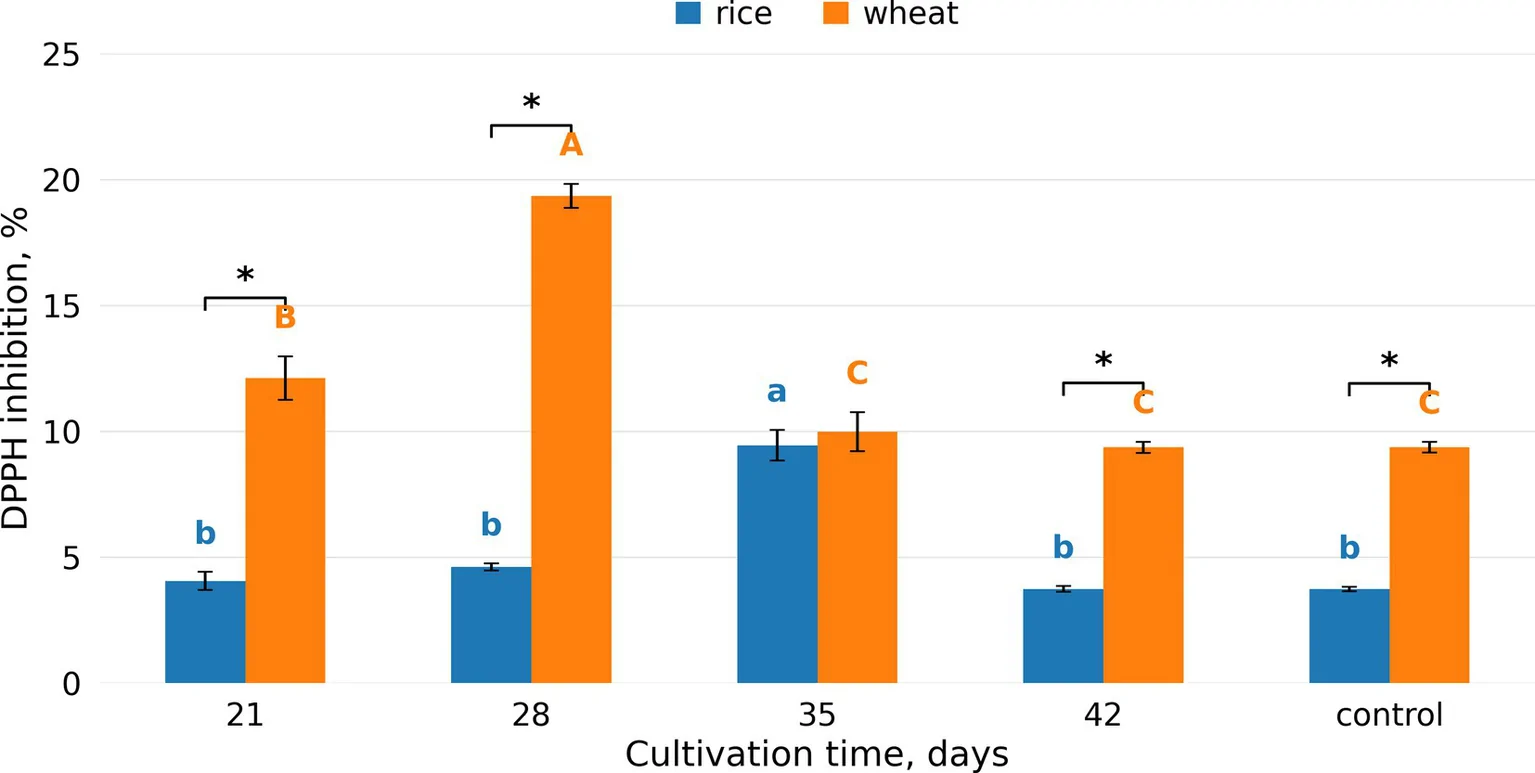
Effect of cultivation time on DPPH inhibition (%) of cereals fermented with *Pleurotus ostreatus* and control (unfermented cereals). Error bars represent mean ± SD of three independent biological replicates (*n* = 3). Different lowercase letters (blue) indicate significant differences among rice cultivation times, whereas different uppercase letters (orange) indicate significant differences among wheat cultivation times, (*) indicates a significant difference between rice and wheat at the same cultivation time (Tukey’s HSD, *p* < 0.05).

Analysis of cereals fermented with *P. ostreatus* highlighted distinct patterns of DPPH radical scavenging activity (Figure 3). The overall trend observed for TPC was reflected in the DPPH inhibition assays. Wheat exhibited higher DPPH radical scavenging activity than rice on days 21, 28, and 42, whereas no significant difference between substrates was observed on day 35. Compared with the unfermented control, DPPH radical scavenging activity was significantly higher on days 21 and 28 in wheat, whereas in rice a significant increase was observed only on day 35. In wheat, the highest DPPH radical scavenging activity coincided with the highest TPC, whereas in rice the highest DPPH activity was observed during the late fermentation stage, when TPC reached its highest numerical values. The highest DPPH radical scavenging activity was observed on day 28 in wheat and on day 35 in rice, corresponding to the time points at which each substrate reached its highest antioxidant activity.

### Multivariate analysis of biochemical changes

**Figure 4:**
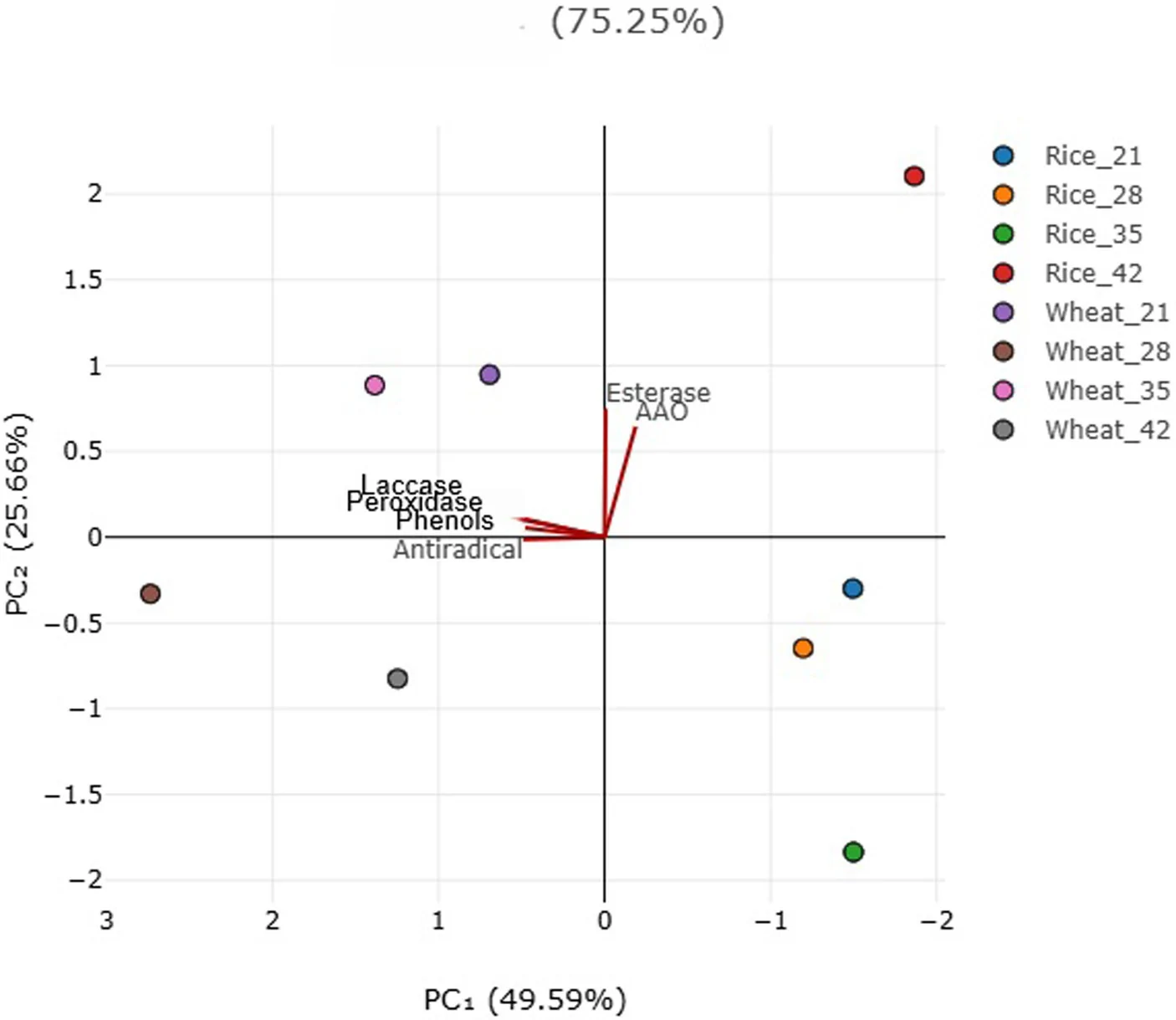
PCA biplot of fermented rice and wheat based on antiradical activity, total phenolic content, and enzyme activities [laccase, peroxidase, esterase, and aryl-alcohol oxidase (AAO)].

Principal component analysis (PCA) was performed to identify relationships among antioxidant activity, TPC, and enzyme activities in fermented rice and wheat samples collected at different time points during cultivation (Figure 4). The first two principal components explained 75.25% of the total variance, indicating that these components captured most of the variability among the samples. Sample distribution indicated that both substrate type and cultivation time contributed to the observed variation. TPC, DPPH radical scavenging activity, and AAO activity clustered together, indicating similar changes during fermentation. In contrast, esterase activity formed a separate group, whereas laccase and peroxidase activities clustered together, suggesting coordinated variation in oxidative enzyme activities.

**Figure 5:**
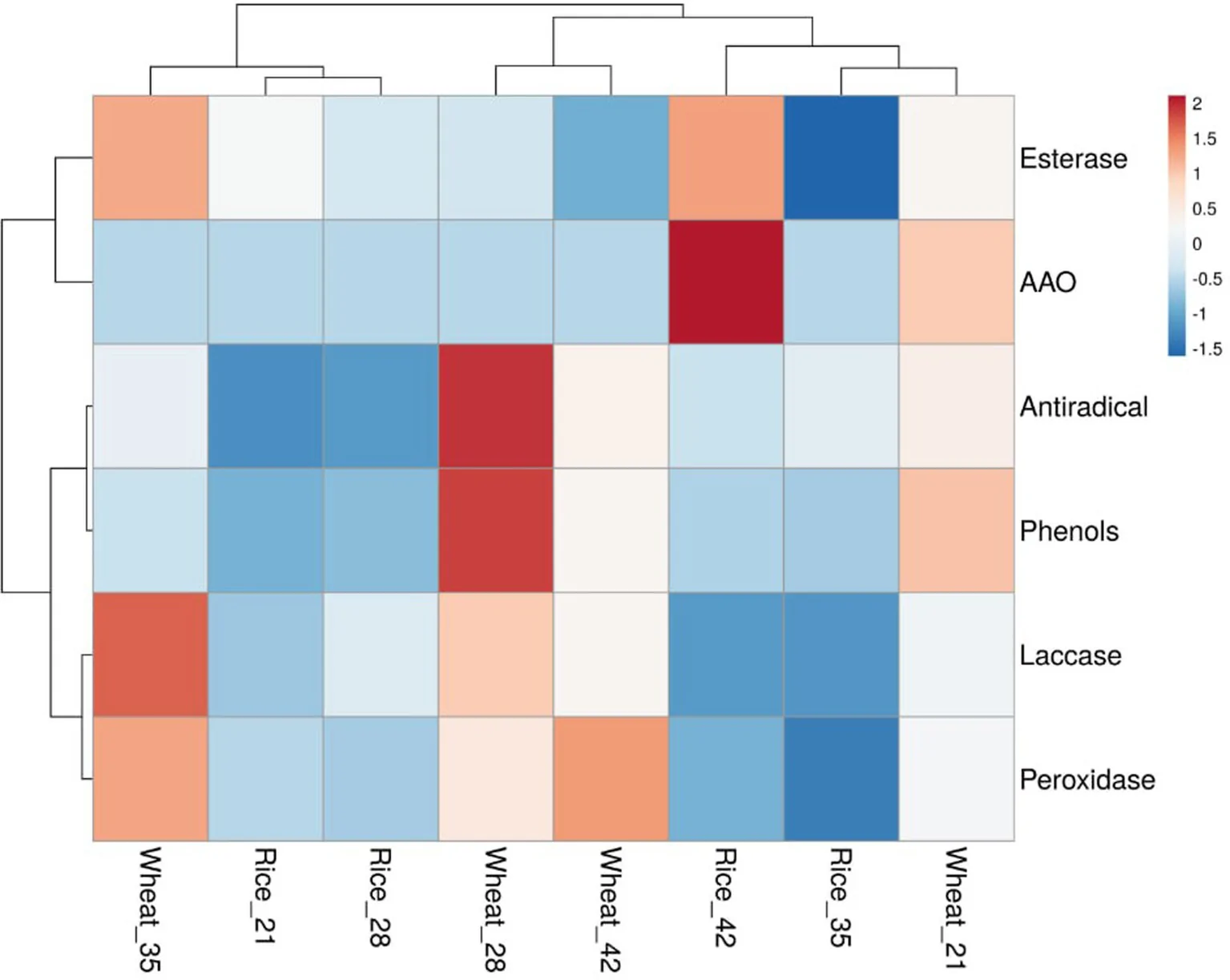
Heatmap and hierarchical clustering of standardized biochemical and enzymatic parameters in fermented rice and wheat samples.

The hierarchical clustering analysis (Figure 5) supported the PCA results and further highlighted the relationships among the measured biochemical parameters. The clustering patterns were consistent with the observed temporal changes in enzyme activities and antioxidant-related parameters. TPC and DPPH radical scavenging activity clustered together and showed similar trends across the samples. Laccase and peroxidase activities were also closely associated, while esterase activity followed a different pattern. AAO was grouped between the antioxidant-related parameters and the oxidative enzymes. Consistent with the PCA, rice and wheat samples were not completely separated.

### Changes in the proximate composition of fermented wheat

Based on the results of our previous screening study (6), wheat was identified as the most suitable substrate for fungal fermentation because of its superior sensory quality and technological performance. Together with the higher antioxidant activity and total phenolic content observed in the present study, these findings supported the selection of wheat for further nutritional characterization, including proximate composition and amino acid analysis. Changes in its proximate chemical composition during cultivation are presented in Table 1. True protein content increased from 6.43 g/100 g DM in the control to 18.65 g/100 g DM after 42 days of cultivation. Ash content also increased over time, whereas carbohydrate content gradually decreased. Fat content showed only minor changes throughout the cultivation period.

**Table 1:** Changes in the proximate chemical composition of wheat during solid-state fermentation with *Pleurotus ostreatus*.

Parameter, g/100 g DM	Control	21 days	28 days	35 days	42 days
True protein	6.43 ± 0.00	8.14 ± 0.01	10.08 ± 0.48	10.45 ± 0.20	18.65 ± 0.25
Fat	1.85 ± 0.05	2.08 ± 0.02	1.54 ± 0.32	1.33 ± 0.44	1.76 ± 0.05
Ash	1.09 ± 0.03	1.36 ± 0.28	1.42 ± 0.16	1.8 ± 0.07	2.16 ± 0.03
Carbohydrates	90.63 ± 0.13	88.42 ± 0.08	86.96 ± 0.59	86.42 ± 0.76	77.43 ± 1.23

Control—unfermented wheat.

### Changes in amino acid composition and protein quality

**Figure 6:**
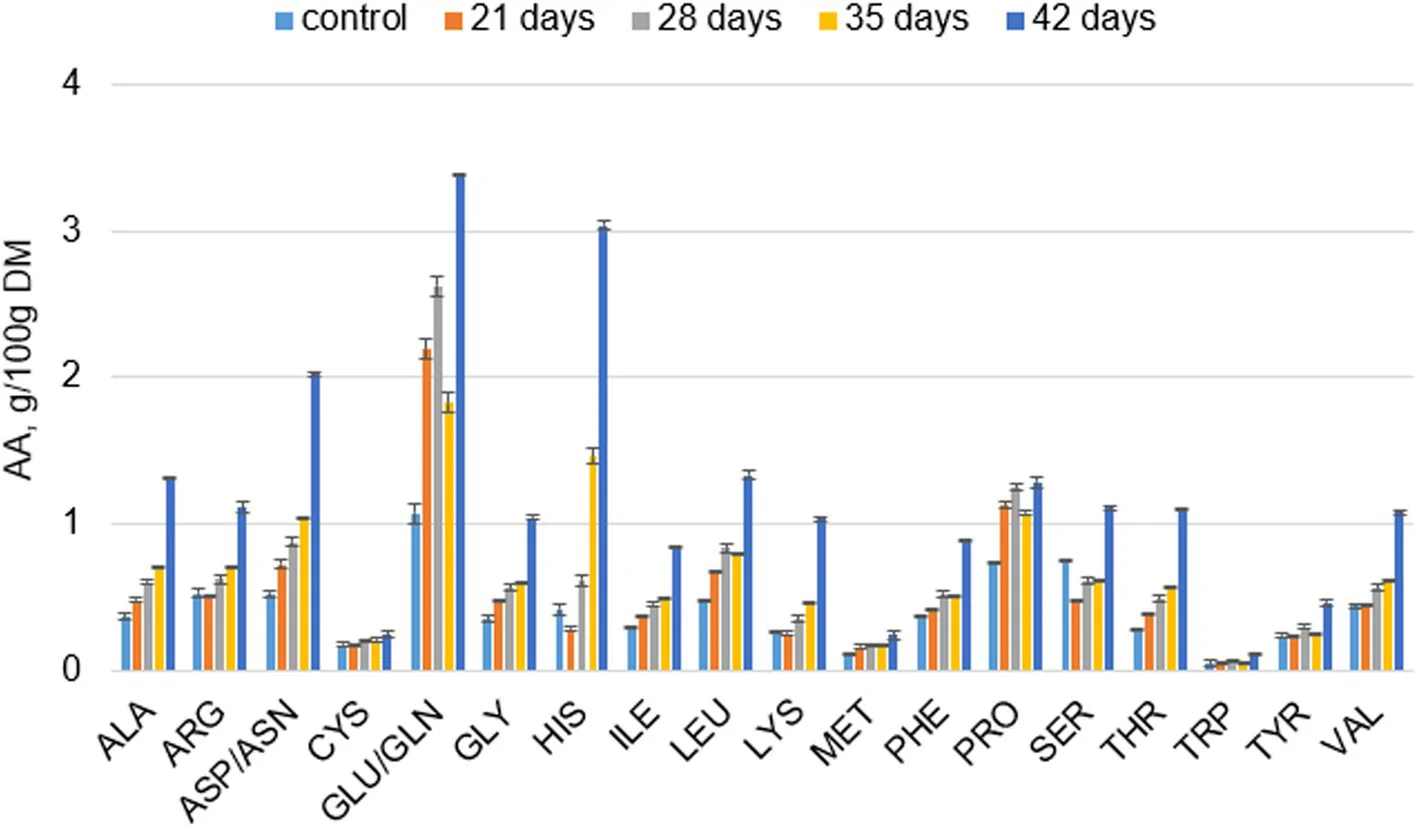
Effect of cultivation time on amino acid composition of wheat fermented with *Pleurotus ostreatus* and control (unfermented wheat). Error bars represent mean ± SD of two independent biological replicates (*n* = 2).

The effect of cultivation time on the amino acid composition of wheat fermented with *P. ostreatus* was evaluated (Figure 6). The highest levels of all analyzed amino acids were observed after 42 days of cultivation. The only exception was proline, for which the values obtained on days 28 and 42 were not significantly different. Most amino acids, including alanine, asparagine/aspartic acid, isoleucine, threonine, and valine, increased progressively throughout cultivation. In contrast, glycine, histidine, proline, and serine showed fluctuations during cultivation. The largest increase from the control to day 42 was observed for histidine, which increased from 0.420 to 3.034 g/100 g DM. Among the essential amino acids, histidine also showed the greatest increase (7.2-fold), followed by threonine (3.9-fold), lysine (3.7-fold), and valine (2.4-fold). The least abundant amino acids after fermentation were tryptophan (0.119 g/100 g DM), methionine (0.245 g/100 g DM), and cysteine (0.249 g/100 g DM).

Changes in the essential amino acid profile and chemical scores during solid-state fermentation of wheat with *P. ostreatus* are presented in Table 2. Tryptophan remained the first limiting amino acid throughout fermentation, whereas lysine was the second limiting amino acid. Threonine and phenylalanine + tyrosine remained at or above the FAO/WHO reference values throughout the cultivation period, while valine reached the reference value on day 35 and remained at or above it thereafter. The chemical scores of leucine declined over time, with the values on days 35 and 42 falling below the FAO/WHO reference value. However, the chemical score of cysteine + methionine met the FAO/WHO reference value only on day 21 of fermentation.

**Table 2:** Changes in essential amino acid composition and chemical scores during solid-state fermentation of wheat with *Pleurotus ostreatus*.

Essential amino acid	Essential amino acid profile (% of total amino acids) and chemical score (CS, %)				
	FAO/WHO (1973) reference values^*^	21 days	28 days	35 days	42 days
Isoleucine	4.0	3.9 (97.5)	3.8 (95)	4.1 (100)	3.9 (97.5)
Leucine	7.0	7.2 (100)	7.1 (100)	6.5 (92.8)	6.1 (87.1)
Lysine	5.5	2.7 (49.1)	3.0 (54.5)	3.8 (69.1)	4.8 (87.3)
Threonine	4.0	4.0 (100)	4.2 (100)	4.7 (100)	5.1 (100)
Tryptophan	1.0	0.6 (60)	0.6 (60)	0.4 (40)	0.5 (50)
Valine	5.0	4.8 (96)	4.8 (96)	5.1 (100)	5.0 (100)
Cysteine + methionine	3.5	3.7 (100)	3.1 (88.6)	3.1 (88.6)	2.3 (65.7)
Phenylalanine + tyrosine	6.0	7.0 (100)	7.0 (100)	6.3 (100)	6.2 (100)

Values in parentheses indicate the chemical score (CS, %). ^*^Chemical scores were calculated according to the FAO/WHO (1973). Values above 100% are reported as 100%.

Table 3 summarizes the effect of cultivation time on amino acid composition and protein quality parameters of wheat fermented with *P. ostreatus*. Compared with the unfermented control, total amino acid content (TAAs) increased throughout cultivation, reaching its highest value on day 42. The proportion of essential amino acids (EAA/Total AA) also increased after an initial decrease on day 21, whereas the essential amino acid index (EAAI) and biological value (BV) decreased relative to the control and then varied only slightly throughout the remaining cultivation period.

**Table 3:** Effect of cultivation time on amino acid protein quality parameters in wheat fermented with *Pleurotus ostreatus*.

Cultivation time, days	TAAs (g/100 g DM)	EAA/total AA (%)	EAAI	BV
0	7.50 ± 0.00	36.51	91.53 ± 0.28	88.07 ± 0.31
21	9.49 ± 0.01	32.26	84.89 ± 0.10	80.83 ± 0.11
28	11.74 ± 0.39	34.75	85.66 ± 0.00	81.67 ± 0.00
35	12.17 ± 0.16	42.24	83.55 ± 1.54	79.37 ± 1.67
42	21.66 ± 0.21	44.69	84.58 ± 1.55	80.50 ± 1.68

TAAs (g/100 g DM), total amino acid content (g/100 g dry matter); EAA, еssential amino acid content, EAA was calculated using the current classification of histidine as an essential amino acid; EAAI, essential amino acid index; and BV, biological value. Values are presented as mean ± SD of two independent biological replicates (*n* = 2).

## Discussion

The temporal changes in enzyme activities provide insight into the dynamics of substrate degradation during solid-state fermentation. Monitoring esterase, laccase, peroxidase, and aryl-alcohol oxidase activities from day 21 to day 42 enabled characterization of the enzymatic dynamics associated with substrate bioconversion. The observed enzyme activity profiles differed between wheat and rice, suggesting that substrate composition influences the enzymatic response of *P. ostreatus*.

In wheat, aryl-alcohol oxidase activity reached its maximum on day 21, whereas the highest laccase and esterase activities were observed on day 35. Peroxidase activity reached its highest numerical value on day 42, although it did not differ significantly from day 35. The early increase in aryl-alcohol oxidase may be related to its role in hydrogen peroxide production, which may contribute to peroxidase-catalyzed reactions. The increase in laccase, peroxidase, and esterase activities at later stages of fermentation is consistent with the degradation of more complex cell wall components after the more readily available nutrients have been utilized. Together, these temporal changes suggest a sequential pattern of substrate degradation during fermentation.

A different pattern was observed in rice. Aryl-alcohol oxidase and esterase activities reached their highest levels on day 42, whereas laccase and peroxidase activities peaked on days 28 and 21, respectively. The contrasting temporal profiles observed in wheat and rice suggest a substrate-dependent enzymatic response, possibly reflecting differences in nutrient availability during fermentation. Wheat and rice differ in cell wall structure and phenolic composition (26, 27), which can influence substrate accessibility and the sequence of polymer degradation. As a result, the timing of enzyme production may vary depending on the structural characteristics of the cereal substrate. Previous studies have also shown that the production of ligninolytic enzymes by *P. ostreatus* depends on cultivation conditions, including substrate composition and growth medium (28–31).

To the best of our knowledge, this is the first study to characterize temporal changes in esterase, aryl-alcohol oxidase, peroxidase, and laccase activities during the solid-state fermentation of wheat and rice by *P. ostreatus*. Most studies have focused on ligninolytic enzyme production during the fermentation of lignocellulosic agro-residues rather than edible cereal substrates (32–35).

Fungal enzymes play an important role in the release and transformation of phenolic compounds during fermentation (36). The observed changes in phenolic content may be explained, at least in part, by the release of bound phenolic compounds following enzymatic modification of cereal cell wall structures, although these processes were not directly examined in the present study. As a result, the measured phenolic content depends not only on the release of bound phenolics but also on their subsequent transformation during fermentation. This interpretation is consistent with the results of the present study. In wheat, the highest total phenolic content and DPPH radical scavenging activity were observed on day 28, whereas laccase, peroxidase, and esterase activities reached their maximum on day 35. In rice, radical scavenging activity was highest on day 35, whereas total phenolic content showed no significant changes throughout the fermentation period, despite slight numerical increases toward days 35–42. These findings suggest that phenolic compounds undergo continuous release and transformation during fermentation. The observed changes in phenolic content and antioxidant activity in our study may reflect the combined action of multiple enzymes rather than the activity of a single enzyme (37). Previous studies have shown that solid-state fermentation with edible and medicinal mushrooms can improve the antioxidant properties and increase the phenolic content of a wide range of plant substrates. Similar effects have been reported for soybean fermented with *Irpex lacteus*, *G. lucidum*, *Pleurotus cornucopiae*, *P. ostreatus*, and *Tricholoma matsutake* (14, 38–41). Increases in antioxidant activity and phenolic content have also been described for fermented rice, wheat, kidney beans, oats, and other cereals fermented with *P. ostreatus*, *Ganoderma sessile*, *A. bisporus, H. lacunosа, F. yanbeiensis* (4, 42, 43). Most of these studies focused on the final changes in phenolic content and antioxidant activity. However, little is known about how fungal enzyme activities are associated with changes in phenolic compounds and antioxidant properties during cereal fermentation. Some studies have investigated the dynamics of phenolic content and antioxidant activity of fermented cereals during fermentation. Lee et al. (41) reported that total phenolic content and radical scavenging activity (DPPH activity) increased with fermentation time during solid-state fermentation of soybean by *T. matsutake* mycelia. A similar finding was reported earlier by Хu et al. (4) during solid-state fermentation of soybean by *A. bisporus*, *H. lacunosa*, and *F. yanbeiensis* mycelia. In contrast, Shon (44) observed a sharp increase in DPPH activity during the early stages of fermentation of beans with *T. matsutake*. However, Kim et al. (38) also reported that DPPH radical scavenging activity changed during soybean fermentation with *I. lacteus* mycelia. Mazzola et al. (43) also suggested that many bioactive compounds are intermediate products of fungal metabolism and therefore may not accumulate continuously during fermentation. This is consistent with our observations that antioxidant activity and, in wheat, total phenolic content varied during fermentation. Previous studies have shown that fungal solid-state fermentation can increase the availability of cereal phenolics through enzymatic modification of the substrate, which may contribute to the functional properties of fermented foods. The higher TPC and DPPH radical scavenging activity observed in fermented wheat suggest that *P. ostreatus* enhanced antioxidant-related properties of the cereal substrate. These findings suggest that fungal fermentation may contribute to favorable changes in the functional characteristics of cereal-based ingredients. However, the present study was limited to biochemical analyses, and further *in vitro* and *in vivo* studies are required to confirm any health-related effects.

The Folin–Ciocalteu assay provides an estimate of the overall reducing capacity of the extracts rather than a specific measure of phenolic compounds. Likewise, antioxidant activity in the present study was evaluated using only the DPPH assay. Therefore, the observed changes should be interpreted as evidence of changes in overall antioxidant-related properties rather than changes in individual phenolic compounds. Future studies combining complementary antioxidant assays with chromatographic profiling of individual phenolic compounds would provide a more comprehensive understanding of the biochemical mechanisms underlying the observed changes during fermentation.

Monitoring fermentation at several time points also provided information that could not be obtained from endpoint measurements alone. The peak enzyme activities, phenolic content, antioxidant activity, and changes in nutritional parameters occurred at different stages of cultivation. These temporal differences demonstrate that substrate transformation is a dynamic process and that the optimal fermentation time depends on the target property.

The PCA and hierarchical clustering analyses revealed similar relationships among the samples and measured parameters. The separation of rice and wheat samples in the PCA plot suggests that substrate composition influenced the observed fermentation profiles. Differences among cultivation times indicate that the biochemical profile of the samples changed throughout the fermentation process. TPC and DPPH radical scavenging activity were closely grouped, indicating a strong association between these parameters. Laccase and peroxidase activities were also clustered together and showed similar trends during fermentation. In contrast, esterase activity showed a distinct pattern compared with the other measured parameters. This difference may indicate that esterase activity responded differently to substrate transformation than the other enzymes examined. Although the hierarchical clustering analysis did not completely separate rice and wheat samples, both the PCA and HCA indicated that substrate type and cultivation time contributed to the observed biochemical variation. This finding suggests that both substrate type and cultivation time contributed to the observed biochemical variation in the fermented materials.

The detailed nutritional characterization focused on wheat following the stepwise evaluation of cereal substrates. In our previous screening study, wheat and rice were identified as the most promising substrates, with wheat showing superior sensory characteristics and greater potential for the development of fermented cereal products. In the present study, wheat again demonstrated higher antioxidant activity and TPC than rice. Together, these findings supported the selection of wheat for detailed nutritional characterization.

In addition to the biochemical changes described above, solid-state fermentation also influences the nutritional composition of cereal grains. Fungal growth and metabolism modify the chemical composition of the substrate and can affect its nutritional quality. In our study, fermentation of wheat with *P. ostreatus* was associated with an increase in true protein content, possibly due to fungal biomass accumulation and carbohydrate utilization during fermentation. Most studies have focused on the overall improvement of nutritional quality by comparing the initial and final stages of fermentation, while temporal changes during the fermentation process have received relatively little attention. Pascual et al. (9) monitored changes in the nutritional and non-nutritional composition of wheat grain and soybeans fermented with *P. ostreatus* over time. They reported improvements in the proximate composition of both fermented substrates, characterized by a linear increase in protein content and a corresponding linear decrease in carbohydrate content. Similar trends were observed in our study. Several studies have reported an increase in protein content following solid-state fermentation with *P. ostreatus*, including in lentils (45) and quinoa (46). In contrast, Ayllón-Parra et al. (47) observed a decrease in the total protein content after SSF of quinoa flour, chickpea flour, oat flour and a blend of 50% (w/w) *Chlorella vulgaris* “Honey” with oat flour with *P. ostreatus*. The lack of significant changes in fat content observed in the present study is consistent with previous reports on solid-state fermentation with *P. ostreatus* (9, 45).

An important finding of the present study is the increase in true protein content and the modification of the amino acid profile during solid-state fermentation of wheat with *P. ostreatus*. The observed increase in amino acid content may be associated with fungal biomass accumulation and protein biosynthesis during fermentation. Among all analyzed amino acids, the largest increase was observed for histidine, whose content increased throughout fermentation and reached a level approximately 7.2-fold higher than that of the control on day 42. Although histidine is not consistently reported as the predominant amino acid in *P. ostreatus* fruiting bodies, recent studies have identified histidine among the major amino acids, together with glutamic and aspartic acids (48). These findings indicate that its abundance may depend on both the cultivation substrate and the physiological state of the fungal biomass. The marked increase in histidine may therefore reflect fungal growth and protein biosynthesis during fermentation. In addition, histidine is the direct precursor of ergothioneine biosynthesis in *P. ostreatus* (49) and other mushrooms (50). Although ergothioneine was not quantified in the present study, the increase in histidine may be consistent with metabolic processes associated with fungal growth and secondary metabolism. Particular attention should be paid to lysine, whose content increased progressively throughout fermentation. Although tryptophan remained the first limiting amino acid throughout the cultivation period, the increase in lysine is nutritionally important. Because lysine is commonly the limiting amino acid in cereal proteins (51, 52), this change is likely to contribute to the improved nutritional quality of fermented wheat. Fermentation also improved the chemical scores of several essential amino acids, particularly lysine. Among the fermented samples, day 28 showed the most balanced essential amino acid profile. Although further fermentation improved the chemical scores of lysine and valine, it was also associated with a decline in tryptophan and sulfur-containing amino acids.

Our results are consistent with previous studies. Lee et al. (41) also reported that prolonged fermentation with *T. matsutake* mycelia was associated with an overall increase in free amino acid content, although the magnitude of the increase varied among individual amino acids. Although Lee et al. (41) reported substantial increases in all branched-chain amino acids, only valine increased significantly in the present study, whereas leucine remained relatively stable and isoleucine showed only minor changes. Similar to their findings, lysine exhibited one of the largest increases during fermentation, highlighting the potential of fungal fermentation to improve the nutritional quality of cereal proteins. Kim and Kim (53) observed changes in the free amino acid profile during soybean fermentation with *I. lacteus* compared with the unfermented substrate. Changes in amino acid composition following solid-state fermentation with *P. ostreatus* have also been reported for alternative plant protein ingredients (47). Consistent with our findings, increases in several essential amino acids, including valine and threonine, were observed, although the magnitude of these changes depended on the substrate.

In addition to the changes observed for individual amino acids and chemical scores, the protein quality indices presented in Table 3 provide additional insight into the nutritional changes occurring during fermentation. Although the total amino acid content increased markedly throughout cultivation, reaching its highest value on day 42, the essential amino acid index (EAAI) and biological value (BV) remained below those of the unfermented control.

These results indicate that fermentation primarily increased the amount of protein-associated amino acids and improved selected amino acid scores rather than the overall balance of essential amino acids. Nevertheless, the proportion of essential amino acids increased during the later stages of fermentation, suggesting a gradual improvement in the nutritional composition of the fermented protein. The relatively small changes in EAAI and BV despite the substantial increase in total amino acid content are consistent with the persistence of limiting amino acids, particularly tryptophan and sulfur-containing amino acids, throughout fermentation. These findings highlight the potential of mycelial fermentation as a sustainable approach for improving the nutritional and functional properties of cereal grains. They also show that fermentation time can be adjusted to obtain products with properties suited to different food applications. Future studies combining multiple antioxidant assays and chromatographic characterization of individual phenolic compounds would provide a more comprehensive understanding of the biochemical changes occurring during fermentation. Future research should also address sensory properties, protein digestibility, consumer acceptance, technological functionality, and scale-up for industrial production, as these aspects were beyond the scope of the present study. Another limitation of the present study is that only an initial (day 0) non-inoculated control was included, whereas time-matched non-inoculated controls at each sampling point were not evaluated. Although many of the observed changes are likely associated with fungal fermentation, a contribution from physicochemical changes during incubation cannot be entirely excluded.

## Conclusion

The present study demonstrated substrate-dependent changes in enzyme activities, total phenolic content, and antioxidant activity in both wheat and rice during *P. ostreatus* solid-state fermentation. Detailed nutritional characterization was performed for wheat because of its higher antioxidant activity and total phenolic content. Different quality parameters peaked at different stages of fermentation, indicating that no single fermentation period is optimal for all product characteristics.

Unlike previous studies focused primarily on endpoint measurements, the time-course approach used here demonstrated that different nutritional, enzymatic, and antioxidant-related properties reached their maxima at different stages of fermentation.

In wheat, the highest total phenolic content and antioxidant activity were observed on day 28, whereas esterase and laccase activities peaked on day 35. Peroxidase activity was numerically highest on day 42 but did not differ significantly from day 35. In rice, laccase, peroxidase, and esterase activities peaked on days 28, 21, and 42, respectively. Total phenolic content reached its highest numerical values during the late fermentation stage (days 35–42) but did not change significantly during fermentation, whereas antioxidant activity peaked on day 35. Aryl-alcohol oxidase activity remained low throughout fermentation in both substrates.

In wheat, nutritional composition changed during the fermentation, with an increase in true protein content and modifications to the essential amino acid profile. Fermentation improved the chemical scores of selected amino acids, particularly lysine, whereas the overall protein quality indices (EAAI and BV) remained below those of the unfermented control. Among the fermented samples, day 28 provided the most balanced amino acid profile, while true protein content continued to increase throughout the cultivation period.

The results show that the optimal fermentation period depends on the desired characteristics of the final product. Monitoring changes throughout fermentation provides a more comprehensive understanding of the process than comparing only the initial and final stages and can guide the selection of fermentation conditions for specific nutritional or functional goals.

This study focused on biochemical and nutritional changes during fermentation and did not examine the molecular mechanisms underlying these changes. Future studies should investigate these mechanisms and their regulation during fermentation.

These findings highlight the potential of *P. ostreatus* solid-state fermentation as an approach for producing cereal ingredients with favorable nutritional and antioxidant-related properties. The time-course analysis provides practical guidance for selecting the optimal fermentation period depending to the intended application and desired characteristics of the final product and may support the development of optimized fermentation processes for functional food applications.

## Data Availability

The original contributions presented in the study are included in the article/Supplementary material, further inquiries can be directed to the corresponding author.

## Footnotes

1 https://biit.cs.ut.ee/clustvis/.
