# Supplementary material for: Changes in nutritional composition and bioactivity of fermented cereals under Pleurotus ostreatus solid-state fermentation
Source: Front Nutr. 2026 Sep 8;13:1919003. doi: 10.3389/fnut.2026.1919003 (PMC13593752; doi:10.3389/fnut.2026.1919003)
Supplement: Supplementary file 1 [file Data_Sheet_1.docx]

**Supplementary Dataset.** Raw replicate data and statistical analyses supporting Figures 1–3 and Table 1.

**Raw data for Figure 1.** Effect of cultivation time on enzymatic activity of cereals fermented with *Pleurotus ostreatus*

| Sample | Esterase | Laccase | Peroxidase | Aryl-alcohol oxidase |
| --- | --- | --- | --- | --- |
| 21rice | 3176.82±150.96 | 262.51±47.35 | 631.75±88.94 | 0 |
| 28 rice | 2624.79±85.87 | 438.69±63.42 | 566.49±35.64 | 1.68±0.29 |
| 35 rice | 1276.79±60.84 | 85.68±22.04 | 131.98±32.93 | 0 |
| 42 rice | 4264.66±97.12 | 102.72±23.03 | 397.05±65.19 | 240.99±5.01 |
| 21wheat | 3240.27±56.34 | 534.98±49.92 | 1033.95±40.70 | 137.23±7.86 |
| 28wheat | 2595.178±92.62 | 847.80±33.76 | 1245.50±55.17 | 0 |
| 35wheat | 4189.23±22.26 | 1095.52±32.26 | 1644.80±33.12 | 0 |
| 42wheat | 1995.91±73.35 | 598.15±29.06 | 1691.66±35.94 | 0 |

**Raw data for Esterase**

**Tukey HSD**

| Tukey HSD | 256,73829 |
| --- | --- |
| k | 8 |
| df | 16 |
| MS within | 8249,349904 |
| n | 3 |
| q crit | 4,896 |

| comparison | absolute difference | critical value | result |
| --- | --- | --- | --- |
| Rice day 21 vs Rice day 28 | 552,03 | 256,74 | means are significantly different |
| Rice day 21 vs Rice day 35 | 1900,03 | 256,74 | means are significantly different |
| Rice day 21 vs Rice day 42 | 1087,84 | 256,74 | means are significantly different |
| Rice day 28 vs Rice day 35 | 1348 | 256,74 | means are significantly different |
| Rice day 28 vs Rice day 42 | 1639,87 | 256,74 | means are significantly different |
| Rice day 35 vs Rice day 42 | 2987,87 | 256,74 | means are significantly different |
| Wheat day 21 vs Wheat day 28 | 645,09 | 256,74 | means are significantly different |
| Wheat day 21 vs Wheat day 35 | 948,96 | 256,74 | means are significantly different |
| Wheat day 21 vs Wheat day 42 | 1244,36 | 256,74 | means are significantly different |
| Wheat day 28 vs Wheat day 35 | 1594,05 | 256,74 | means are significantly different |
| Wheat day 28 vs Wheat day 42 | 599,27 | 256,74 | means are significantly different |
| Wheat day 35 vs Wheat day 42 | 2193,32 | 256,74 | means are significantly different |
| Rice day 21 vs Wheat day 21 | 63,45 | 256,74 | means are not signigicantly different |
| Rice day 28 vs Wheat day 28 | 29,61 | 256,74 | means are not signigicantly different |
| Rice day 35 vs Wheat day t35 | 2912,44 | 256,74 | means are significantly different |
| Rice day 42 vs Wheat day 42 | 2268,75 | 256,74 | means are significantly different |

**Raw data for Laccase**

**Tukey HSD**

| Tukey HSD | 112,4213774 |
| --- | --- |
| k | 8 |
| df | 16 |
| MS within | 1581,744163 |
| n | 3 |
| q crit | 4,896 |

| comparison | absolute difference | critical value | result |
| --- | --- | --- | --- |
| Rice day 21 vs Rice day 28 | 176,18 | 112,42 | means are significantly different |
| Rice day 21 vs Rice day 35 | 176,83 | 112,42 | means are significantly different |
| Rice day 21 vs Rice day 42 | 159,79 | 112,42 | means are significantly different |
| Rice day 28 vs Rice day 35 | 353,01 | 112,42 | means are significantly different |
| Rice day 28 vs Rice day 42 | 335,97 | 112,42 | means are significantly different |
| Rice day 35 vs Rice day 42 | 17,04 | 112,42 | means are not signigicantly different |
| Wheat day 21 vs Wheat day 28 | 312,82 | 112,42 | means are significantly different |
| Wheat day 21 vs Wheat day 35 | 560,54 | 112,42 | means are significantly different |
| Wheat day 21 vs Wheat day 42 | 63,17 | 112,42 | means are not signigicantly different |
| Wheat day 28 vs Wheat day 35 | 247,72 | 112,42 | means are significantly different |
| Wheat day 28 vs Wheat day 42 | 249,65 | 112,42 | means are significantly different |
| Wheat day 35 vs Wheat day 42 | 497,37 | 112,42 | means are significantly different |
| Rice day 21 vs Wheat day 21 | 272,47 | 112,42 | means are significantly different |
| Rice day 28 vs Wheat day 28 | 409,11 | 112,42 | means are significantly different |
| Rice day 35 vs Wheat day 35 | 1009,84 | 112,42 | means are significantly different |
| Rice day 42 vs Wheat day 42 | 495,43 | 112,42 | means are significantly different |

**Raw data for Peroxidase**

**Tukey HSD**

| Tukey HSD | 145,7760542 |
| --- | --- |
| k | 8 |
| df | 16 |
| MS within | 2659,566279 |
| n | 3 |
| q crit | 4,896 |

| comparison | absolute difference | critical value | result |
| --- | --- | --- | --- |
| Rice day 21 vs Rice day 28 | 65,26 | 145,78 | means are not signigicantly different |
| Rice day 21 vs Rice day 35 | 499,77 | 145,78 | means are significantly different |
| Rice day 21 vs Rice day 42 | 234,7 | 145,78 | means are significantly different |
| Rice day 28 vs Rice day 35 | 434,51 | 145,78 | means are significantly different |
| Rice day 28 vs Rice day 42 | 169,44 | 145,78 | means are significantly different |
| Rice day 35 vs Rice day 42 | 265,07 | 145,78 | means are significantly different |
| Wheat day 21 vs Wheat day 28 | 211,55 | 145,78 | means are significantly different |
| Wheat day 21 vs Wheat day 35 | 610,85 | 145,78 | means are significantly different |
| Wheat day 21 vs Wheat day 42 | 657,71 | 145,78 | means are significantly different |
| Wheat day 28 vs Wheat day 35 | 399,3 | 145,78 | means are significantly different |
| Wheat day 28 vs Wheat day 42 | 446,16 | 145,78 | means are significantly different |
| Wheat day 35 vs Wheat day 42 | 46,86 | 145,78 | means are not signigicantly different |
| Rice day 21 vs Wheat day 21 | 402,2 | 145,78 | means are significantly different |
| Rice day 28 vs Wheat day 28 | 679,01 | 145,78 | means are significantly different |
| Rice day 35 vs Wheat day t35 | 1512,82 | 145,78 | means are significantly different |
| Rice day 42 vs Wheat day 42 | 1294,61 | 145,78 | means are significantly different |

**Raw data for aryl-alcohol oxidase**

**Tukey HSD**

| Tukey HSD | 9,249602061 |
| --- | --- |
| k | 8 |
| df | 16 |
| MS within | 10,7074125 |
| n | 3 |
| q crit | 4,896 |

| comparison | absolute difference | critical value | result |
| --- | --- | --- | --- |
| Rice day 21 vs Rice day 28 | 1,68 | 9,25 | means are not signigicantly different |
| Rice day 21 vs Rice day 35 | 0 | 9,25 | means are not signigicantly different |
| Rice day 21 vs Rice day 42 | 240,99 | 9,25 | means are significantly different |
| Rice day 28 vs Rice day 35 | 1,68 | 9,25 | means are not signigicantly different |
| Rice day 28 vs Rice day 42 | 239,31 | 9,25 | means are significantly different |
| Rice day 35 vs Rice day 42 | 240,99 | 9,25 | means are significantly different |
| Wheat day 21 vs Wheat day 28 | 137,23 | 9,25 | means are significantly different |
| Wheat day 21 vs Wheat day 35 | 137,23 | 9,25 | means are significantly different |
| Wheat day 21 vs Wheat day 42 | 137,23 | 9,25 | means are significantly different |
| Wheat day 28 vs Wheat day 35 | 0 | 9,25 | means are not signigicantly different |
| Wheat day 28 vs Wheat day 42 | 0 | 9,25 | means are not signigicantly different |
| Wheat day 35 vs Wheat day 42 | 0 | 9,25 | means are not signigicantly different |
| Rice day 21 vs Wheat day 21 | 137,23 | 9,25 | means are significantly different |
| Rice day 28 vs Wheat day 28 | 1,68 | 9,25 | means are not signigicantly different |
| Rice day 35 vs Whea day t35 | 0 | 9,25 | means are not signigicantly different |
| Rice day 42 vs Wheat day 42 | 240,99 | 9,25 | means are significantly different |

**Raw data for Figure 2**. Effect of cultivation time on total phenolic content of cereals fermented with *Pleurotus ostreatus* and control (unfermented cereals).

| Sample | rice | wheat |
| --- | --- | --- |
| 0 (control) | 0.02±0.00 | 0.04±0.02 |
| 21 | 0.03±0.01 | 0.42±0.03 |
| 28 | 0.05±0.02 | 0.59±0.07 |
| 35 | 0.08±0.01 | 0.03±0.00 |
| 42 | 0.10±0.04 | 0.27±0.06 |

**Raw data for total phenolic content of cereals**

**Tukey HSD**

| Tukey HSD | 0,103764879 |
| --- | --- |
| k | 10 |
| df | 20 |
| MS within | 0,001287933 |
| n | 3 |
| q crit | 5,008 |

| comparison | absolute difference | critical value | result |
| --- | --- | --- | --- |
| Rice day 0 vs Rice day 21 | 0,01 | 0,10 | means are not signigicantly different |
| Rice day 0 vs Rice day 28 | 0,03 | 0,10 | means are not signigicantly different |
| Rice day 0 vs Rice day 35 | 0,06 | 0,10 | means are not signigicantly different |
| Rice day 0 vs Rice day 42 | 0,08 | 0,10 | means are not signigicantly different |
| Rice day 21 vs Rice day 28 | 0,02 | 0,10 | means are not signigicantly different |
| Rice day 21 vs Rice day 35 | 0,05 | 0,10 | means are not signigicantly different |
| Rice day 21 vs Rice day 42 | 0,07 | 0,10 | means are not signigicantly different |
| Rice day 28 vs Rice day 35 | 0,03 | 0,10 | means are not signigicantly different |
| Rice day 28 vs Rice day 42 | 0,05 | 0,10 | means are not signigicantly different |
| Rice day 35 vs Rice day 42 | 0,02 | 0,10 | means are not signigicantly different |
| Wheat day 0 vs Wheat day 21 | 0,38 | 0,10 | means are significantly different |
| Wheat day 0 vs Wheat day 28 | 0,55 | 0,10 | means are significantly different |
| Wheat day 0 vs Wheat day 35 | 0,01 | 0,10 | means are not signigicantly different |
| Wheat day 0 vs Wheat day 42 | 0,23 | 0,10 | means are significantly different |
| Wheat day 21 vs Wheat day 28 | 0,17 | 0,10 | means are significantly different |
| Wheat day 21 vs Wheat day 35 | 0,39 | 0,10 | means are significantly different |
| Wheat day 21 vs Wheat day 42 | 0,15 | 0,10 | means are significantly different |
| Wheat day 28 vs Wheat day 35 | 0,56 | 0,10 | means are significantly different |
| Wheat day 28 vs Wheat day 42 | 0,32 | 0,10 | means are significantly different |
| Wheat day 35 vs Wheat day 42 | 0,24 | 0,10 | means are significantly different |
| Rice day 0 vs Wheat day 0 | 0,02 | 0,10 | means are not signigicantly different |
| Rice day 21 vs Wheat day 21 | 0,39 | 0,10 | means are significantly different |
| Rice day 28 vs Wheat day 28 | 0,54 | 0,10 | means are significantly different |
| Rice day 35 vs Wheat day 35 | 0,05 | 0,10 | means are not signigicantly different |
| Rice day 42 vs Wheat day 42 | 0,17 | 0,10 | means are significantly different |

**Raw data for Figure 3.** Effect of cultivation time on DPPH inhibition (%) of cereals fermented with *Pleurotus ostreatus* and control (unfermented cereals).

| Sample | rice | wheat |
| --- | --- | --- |
| control | 3.73±0.10 | 9.36±0.22 |
| 21 | 4.05±0.41 | 12.11±0.90 |
| 28 | 4.61±0.15 | 19.35±0.47 |
| 35 | 9.44±0.61 | 9.98±0.81 |
| 42 | 3.73±0.12 | 9.36±0.24 |

Raw data for DPPH inhibition

**Tukey HSD**

| Tukey HSD | 1,3556179 |
| --- | --- |
| k | 10 |
| df | 20 |
| MS within | 0,21982 |
| n | 3 |
| q crit | 5,008 |

| comparison | absolute difference | critical value | result |
| --- | --- | --- | --- |
| Rice day 0 vs Rice day 21 | 0,32 | 1,36 | means are not signigicantly different |
| Rice day 0 vs Rice day 28 | 0,88 | 1,36 | means are not signigicantly different |
| Rice day 0 vs Rice day 35 | 5,71 | 1,36 | means are significantly different |
| Rice day 0 vs Rice day 42 | 0 | 1,36 | means are not signigicantly different |
| Rice day 21 vs Rice day 28 | 0,56 | 1,36 | means are not signigicantly different |
| Rice day 21 vs Rice day 35 | 5,39 | 1,36 | means are significantly different |
| Rice day 21 vs Rice day 42 | 0,32 | 1,36 | means are not signigicantly different |
| Rice day 28 vs Rice day 35 | 4,83 | 1,36 | means are significantly different |
| Rice day 28 vs Rice day 42 | 0,88 | 1,36 | means are not signigicantly different |
| Rice day 35 vs Rice day 42 | 5,71 | 1,36 | means are significantly different |
| Wheat day 0 vs Wheat day 21 | 2,75 | 1,36 | means are significantly different |
| Wheat day 0 vs Wheat day 28 | 9,99 | 1,36 | means are significantly different |
| Wheat day 0 vs Wheat day 35 | 0,62 | 1,36 | means are not signigicantly different |
| Wheat day 0 vs Wheat day 42 | 0 | 1,36 | means are not signigicantly different |
| Wheat day 21 vs Wheat day 28 | 7,24 | 1,36 | means are significantly different |
| Wheat day 21 vs Wheat day 35 | 2,13 | 1,36 | means are significantly different |
| Wheat day 21 vs Wheat day 42 | 2,75 | 1,36 | means are significantly different |
| Wheat day 28 vs Wheat day 35 | 9,37 | 1,36 | means are significantly different |
| Wheat day 28 vs Wheat day 42 | 9,99 | 1,36 | means are significantly different |
| Wheat day 35 vs Wheat day 42 | 0,62 | 1,36 | means are not signigicantly different |
| Rice day 0 vs Wheat day 0 | 5,63 | 1,36 | means are significantly different |
| Rice day 21 vs Wheat day 21 | 8,06 | 1,36 | means are significantly different |
| Rice day 28 vs Wheat day 28 | 14,74 | 1,36 | means are significantly different |
| Rice day 35 vs Wheat day 35 | 0,54 | 1,36 | means are not signigicantly different |
| Rice day 42 vs Wheat day 42 | 5,63 | 1,36 | means are significantly different |

**Raw data for Table 1.** Fat and ash contents of wheat during solid-state fermentation with Pleurotus ostreatus

| Cultivation time (days) | Fat  (g/100 g DM) | Ash  (g/100 g DM) |
| --- | --- | --- |
| 0 | 1,84 | 1,06 |
| 0 | 1,80 | 1,11 |
| 0 | 1,90 | 1,10 |
| 21 | 2,08 | 1,68 |
| 21 | 2,10 | 1,24 |
| 21 | 2,07 | 1,16 |
| 28 | 1,86 | 1,24 |
| 28 | 1,22 | 1,50 |
| 28 | 1,54 | 1,52 |
| 35 | 1,83 | 1,73 |
| 35 | 1,00 | 1,80 |
| 35 | 1,16 | 1,87 |
| 42 | 1,71 | 2,14 |
| 42 | 1,75 | 2,19 |
| 42 | 1,81 | 2,15 |
